# Acute primary chylous peritonitis mimicking acute abdomen: a case report and literature review

**DOI:** 10.11604/pamj.2024.47.131.42794

**Published:** 2024-03-22

**Authors:** Hanen Zenati, Maissa Jallali, Asma Korbi, Amina Chaka, Sadok Ben Jabra, Ibtissem Korbi, Faouzi Noomen

**Affiliations:** 1 Department of Visceral and Digestive Surgery, University Hospital of Monastir, Monastir, Tunisia,; 2 Department of Gynecology, University Hospital of Monastir, Monastir, Tunisia

**Keywords:** Acute, chylous peritonitis, abdomen, laparotomy, lymphatic fluid, case report

## Abstract

Acute chylous peritonitis is an uncommon medical condition that can occur suddenly, resulting in the buildup of chylous fluid in the peritoneal cavity. It is considered idiopathic because the exact cause is often unknown. The symptoms of acute chylous idiopathic peritonitis can mimic other abdominal emergencies, making it challenging to diagnose and manage, requiring a multidisciplinary approach. We present a case report of acute idiopathic chylous peritonitis miming acute abdomen, how was successfully treated with surgery, and provide a comprehensive review of the available literature on this topic. Chylous peritonitis is a rare condition whose clinical presentation mimics an acute abdomen. It is necessary to undertake careful exploration. An emergent laparotomy is indicated to treat the peritonitis and search for and treat the underlying cause.

## Introduction

Acute chylous peritonitis is an uncommon medical condition characterized by the abrupt accumulation of chylous fluid in the peritoneal cavity, which is rich in triglycerides due to the presence of lymph [1]. This condition usually occurs without any significant underlying pathology, and in over 50% of reported cases, no underlying cause was determined [2]. However, it is difficult to diagnose preoperatively due to its sudden onset and accompanying acute abdomen signs. It is typically identified during laparotomy since there are no distinctive features to suspect this entity. Few instances of acute chylous peritonitis are documented in the literature, with various underlying causes identified. These include abdominal malignancies, cirrhosis, inflammation, congenital factors, post-operative or traumatic origins, and other miscellaneous disorders [3].

In this report, we present a case of a patient with signs of acute abdomen due to acute chylous peritonitis who was successfully treated with surgery.

## Patient and observation

**Patient information:** a 61-year-old male with a medical history of type 2 diabetes mellitus and hyperlipidemia presented to the emergency department with a three-day history of persistent abdominal distension and pain that exacerbated with any movement. The patient's medical background indicated no history of systemic diseases, previous surgeries, or abdominal trauma. Additionally, he reported no alcohol or cigarette consumption and denied recent extensive travel.

**Clinical findings:** during examination, the patient exhibited a temperature of 38.5°C, a pulse rate of 100 beats per minute, and a blood pressure reading of 140/80 mmHg. No significant abnormalities were noted upon systemic assessment. However, the abdomen appeared mildly distended, with hypoactive bowel sounds. Generalized abdominal guarding was also evident.

**Timeline of the current episode:** the patient's symptoms had been evolving for 3 days.

**Figure 1:**
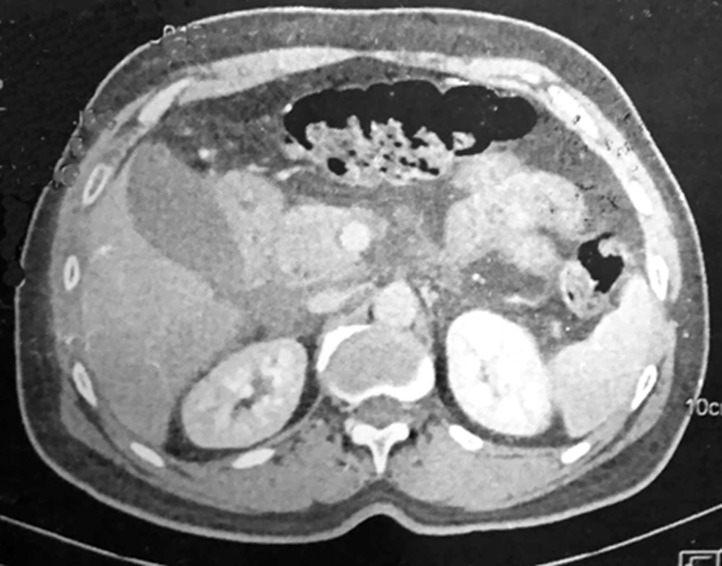
preoperative computed tomography showing intra-abdominal fluid and infiltration of the epigastric fat extending to the hepatic hilum and the root of the mesentery

**Diagnostic assessment:** blood analysis gave the following values: total leukocyte count: 15.2 x 109/L; hemoglobin: 13 g/dL; platelet count: 299 x 109/L; urea: 5.3 mmol/L; creatinine: 48 µmol/L; aspartate aminotransferase: 22 nkat/L; alanine aminotransferase: 22 nkat/L; serum lipase: 37 U/L; CRP: 189 mg/L. A computed tomography scan of the abdomen with contrast showed free fluid and infiltration of the epigastric fat extending to the hepatic hilum and the root of the mesentery, with thickening of the peritoneal layers, and a regular thickening of the antral and pyloric walls extending to the duodenum and proximal jejunum (Figure 1). There were no signs indicating perforation or other surgical pathology.

**Diagnosis:** the patient was diagnosed with acute abdominal emergencies.

**Figure 2:**
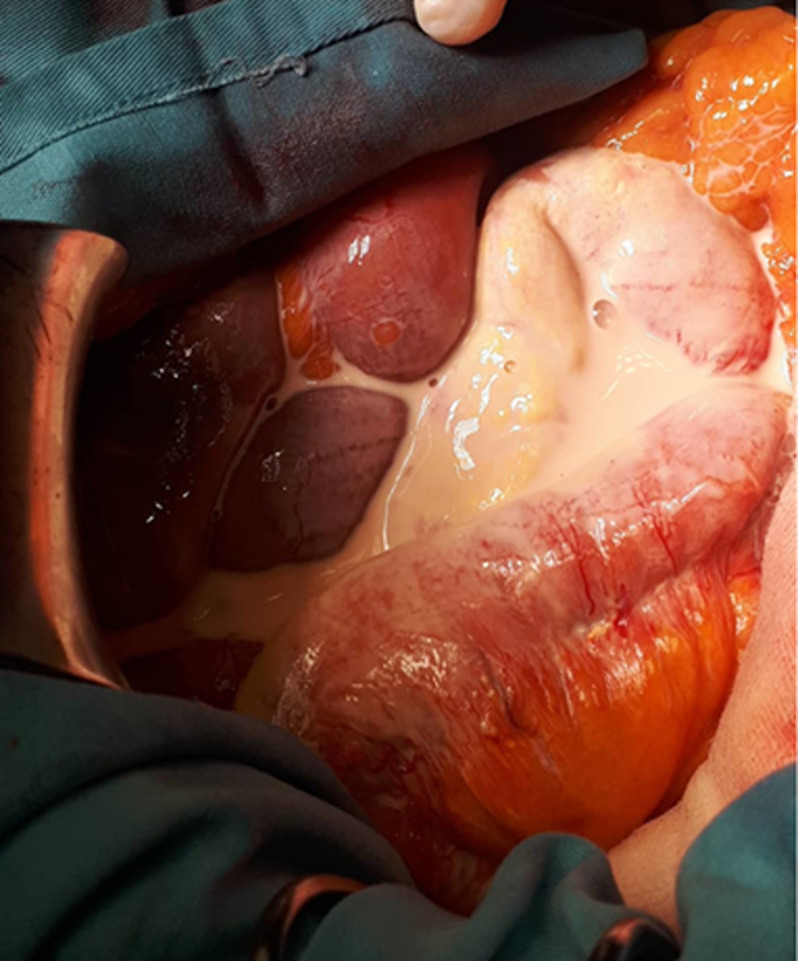
intra-operative view showing the milky-like appearance of the fluid in the abdominal cavity

**Figure 3:**
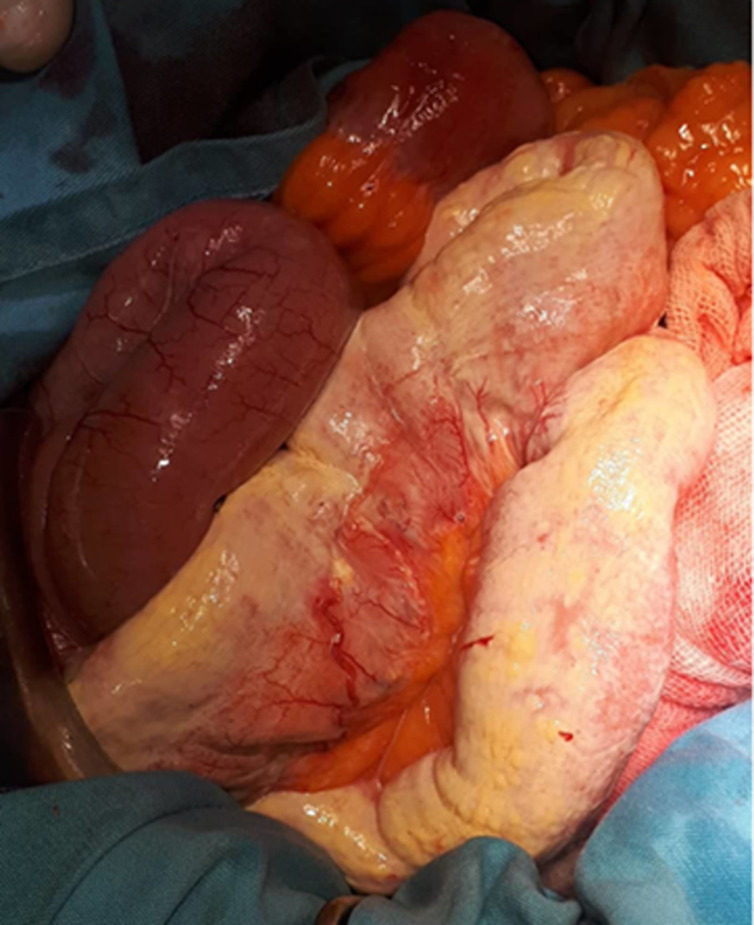
operative view showing the congestive wall of the small bowel due to dilated lymphatics

**Therapeutic interventions:** during the exploratory laparotomy, upon entry into the peritoneum, a significant volume of milky fluid was observed within the peritoneal cavity (Figure 2), associated with a congestive wall of the small bowel due to dilated lymphatics (Figure 3). This observation suggests the presence of chylous fluid, which is a key finding in cases of acute chylous peritonitis. The exploration of the entire abdomen, including examination of intestinal segments, pelvic organs, the appendix, the retroperitoneal area, the posterior side of the stomach, and the pancreas, did not reveal any surgical pathologies that could account for the patient's clinical presentation. This suggests that there were no findings such as perforation, pancreatitis, appendicitis, cholecystitis, ischemia, or diverticulitis that could explain the symptoms experienced by the patient. Despite the thorough exploration, the underlying cause of the acute abdominal symptoms and the accumulation of milky fluid in the peritoneal cavity remained unidentified. The procedure was terminated by peritoneal lavage and drainage, and the fluid was sent for biochemical, cytological, and bacterial analysis. The analysis of the peritoneal liquid showed no malignant cells, negative bacterial cultures, and a triglyceride concentration at ten times the serum level, confirming it to be chyle. The oncological markers (α-fetoprotein, carcino-embryonic antigen, and carbonic anhydrase 19-9) were negative.

**Follow-up and outcome of interventions:** the patient experienced an uneventful postoperative period and demonstrated good recovery following the exploratory laparotomy. Initiating a low-fat diet likely contributed to the patient's improvement. Furthermore, the absence of complications or recurrence during the 36-month follow-up is positive news, suggesting a favorable long-term outcome. This indicates successful management of the acute abdominal condition, although the underlying cause remains unresolved.

**Patient perspective:** the patient was satisfied with symptom improvement and pleased with the surgery.

**Informed consent:** the patient's consent was voluntary and informed.

## Discussion

Chylous peritonitis is a rare condition characterized by the accumulation of chyle in the peritoneal cavity. Chyle, a milky fluid composed of lymph and fat, is typically transported through the lymphatic system. However, in cases of acute chylous peritonitis, there is a disruption in the normal flow of chyle, leading to its abnormal accumulation [1].

Acute chylous peritonitis is an extremely rare condition, with only a handful of cases reported in the literature. The accumulation of triglyceride-rich, milky fluid in the peritoneal cavity, as observed in cases of chylous peritonitis, can be attributed to various disorders. These may include abdominal malignancies, cirrhosis, congenital diseases, and postoperative or traumatic causes [2-4]. However, as described in your case, there are instances where no identifiable underlying cause or disease is evident, and this condition is termed “primary chylous peritonitis”. The exact cause of acute idiopathic chylous peritonitis remains unknown. Some suggest that increased permeability of the lymphatic vessels due to a sudden pressure increase in the lymph canals after a heavy meal may lead to the leakage of chyle into the peritoneal cavity [5]. The symptoms of chylous peritonitis can vary depending on the severity of the condition. Patients with acute idiopathic chylous peritonitis typically present with symptoms and signs suggestive of an acute abdomen. The most common complaints include abdominal pain, tenderness, distension, and peritonism [6,7]. Other features may include nausea, vomiting, early satiety, diarrhea, steatorrhea, malnutrition, edema, fever, and night sweats [3,6]. Physical examination may reveal signs of abdominal distension and tenderness. These symptoms can be misinterpreted as other acute abdominal emergencies, such as acute appendicitis, pancreatitis, or ovarian torsion, making the diagnosis challenging [7]. Laboratory tests can aid in the diagnosis of acute idiopathic chylous peritonitis. Elevated levels of triglycerides and chylomicrons in the peritoneal fluid are commonly observed. Imaging techniques, including ultrasound and computed tomography (CT) scans, are not specific to chylous ascites but are useful in identifying intraabdominal fluid and detecting any underlying causes contributing to the condition [8].

Given the nonspecific nature of the symptoms, clinicians must consider acute chylous peritonitis in the differential diagnosis, especially in cases where there is no clear etiology for the acute abdomen. The diagnosis of acute idiopathic chylous peritonitis is usually made intraoperatively, as there are no specific clinical or imaging findings to confirm the condition preoperatively. Diagnostic procedures such as laparoscopy or laparotomy are often performed due to acute abdominal complaints. During the procedure, the presence of chylous effusion with milky-like fluid in the peritoneal cavity confirms the diagnosis [7].

Biochemical analysis of the peritoneal fluid aids in differentiating chylous peritonitis from other causes of acute abdomen, such as infectious peritonitis or pancreatitis. The triglyceride level of this milky fluid being two to eight times that of the normal plasma triglyceride levels is an important diagnostic feature of chylous ascites.

The management of chylous ascites is a multi-faceted process with limited available therapeutic options. Treatment should be individualized and adjusted according to the severity of chylous ascites. Treatment for acute idiopathic chylous peritonitis aims to alleviate symptoms and prevent complications and recurrences. Successful results have been reported with a conservative approach, including total parenteral nutrition and somatostatin administration in cases where the clinical condition developed slowly [6,7]. The initial step usually involves conservative management and dietary modifications, including a low-fat and high-protein diet. Dietary modifications aim to reduce the production of chyle and alleviate the burden on the lymphatic system. Patients are advised to avoid fatty or high-protein meals and opt for a low-fat diet. Additionally, medium-chain triglyceride (MCT) supplements may be recommended as they are absorbed directly into the bloodstream, bypassing the lymphatic system [7-10].

Draining the excess chyle from the peritoneal cavity is another crucial aspect of treatment. This can be achieved through paracentesis. In severe cases, presenting acute abdomen findings, such as in our case, laparotomy is recommended because it is beneficial to make a correct diagnosis and find the underlying cause. Surgical intervention includes ligation of the damaged lymph canal, ligation of lymph vessels, omental patching, or peritoneovenous suction. If there is no underlying cause of free chylous observed in surgery, aspiration of ascites and abdominal lavage are the therapeutic methods. However, malignant, malabsorption, and infectious predisposing factors must be evaluated even after the operation for any underlying disease that can lead to the formation of chylous ascites. This approach was taken in our case and the patient was tested, released, and followed up to this day without any problems. Laparoscopy is a widely used and recommended procedure in acute abdominal cases where a preoperative-specific diagnosis cannot be made [4,9].

The prognosis for acute idiopathic chylous peritonitis varies depending on the underlying cause and the timeliness of treatment. With appropriate management, many patients experience a resolution of symptoms and a return to normal health. However, in some cases, complications such as chronic chylous peritonitis or recurrent episodes may occur [10].

## Conclusion

Acute chylous peritonitis is a very rare and severe condition that can cause acute abdominal syndrome. To elucidate the etiology, a meticulous exploration should be performed.
